# Effects of gut microbiota on neurodegenerative diseases

**DOI:** 10.3389/fnagi.2023.1145241

**Published:** 2023-05-30

**Authors:** Saima Khatoon, Nida Kalam, Summya Rashid, Gulnaz Bano

**Affiliations:** ^1^Department of Medical Elementology and Toxicology, School of Chemical and Life Sciences, Jamia Hamdard, New Delhi, India; ^2^Department of Pharmacology, School of Pharmaceutical Education and Research, Jamia Hamdard, New Delhi, India; ^3^Department of Pharmacology and Toxicology, College of Pharmacy, Prince Sattam Bin Abdulaziz University, Al-Kharj, Saudi Arabia

**Keywords:** neurodegenerative diseases, gut-brain axis, microbiota, Alzheimer’s disease, Parkinson’s disease, Huntington’s disease

## Abstract

A progressive degradation of the brain’s structure and function, which results in a reduction in cognitive and motor skills, characterizes neurodegenerative diseases (NDs) such as Alzheimer’s disease (AD), Parkinson’s disease (PD), Amyotrophic lateral sclerosis (ALS), and Huntington’s disease (HD). The morbidity linked to NDs is growing, which poses a severe threat to human being’s mental and physical ability to live well. The gut-brain axis (GBA) is now known to have a crucial role in the emergence of NDs. The gut microbiota is a conduit for the GBA, a two-way communication system between the gut and the brain. The myriad microorganisms that make up the gut microbiota can affect brain physiology by transmitting numerous microbial chemicals from the gut to the brain *via* the GBA or neurological system. The synthesis of neurotransmitters, the immunological response, and the metabolism of lipids and glucose have all been demonstrated to be impacted by alterations in the gut microbiota, such as an imbalance of helpful and harmful bacteria. In order to develop innovative interventions and clinical therapies for NDs, it is crucial to comprehend the participation of the gut microbiota in these conditions. In addition to using antibiotics and other drugs to target particular bacterial species that may be a factor in NDs, this also includes using probiotics and other fecal microbiota transplantation to maintain a healthy gut microbiota. In conclusion, the examination of the GBA can aid in understanding the etiology and development of NDs, which may benefit the improvement of clinical treatments for these disorders and ND interventions. This review indicates existing knowledge about the involvement of microbiota present in the gut in NDs and potential treatment options.

## 1. Introduction

The progressive loss of function and death of neurons in the brain and other areas of the nervous system characterizes neurodegenerative diseases (NDs). NDs have been associated with various factors, including genetics, environmental toxins, and abnormal protein aggregation in the brain. Some specific examples of neurotoxic chemicals that have been implicated in the development of NDs include beta-amyloid (Aβ) and tau proteins in Alzheimer’s disease (AD), α-synuclein (α-syn) in Parkinson’s disease (PD), and Huntington protein in Huntington’s disease (HD). The expansion of human life expectancy is attributable to the advancements in diet and healthcare brought by the growth of the economy and technology. With the advancement of age, the prevalence of these diseases rises to place heavier and heavier obligations on society. Unfortunately, despite of numerous current clinical trials, there is no effective treatment for these NDs due to their uncertain origin and the intricacy of the central nervous system (CNS).

The gut microbiota (GM) is a community that is a permanent resident of gastrointestinal tract (GIT). The GM are crucial for maintaining the gut homeostasis and overall well-being. The GM comprises a diverse group of microorganisms, including bacteria, viruses, fungi, and protozoa, which together functions to digest food, produce nutrients, and support the immune system (Arumugam et al., 2011). The GM significantly influences the physiology of the host in many ways, such as neuronal growth, maintaining immunological functions, produce an anti-infection effect, and enhancing nutritional efficiency (Sommer and Bäckhed, 2013; Geva-Zatorsky et al., 2017). There are multiple challenges faced by GM such as changes in food habits like more consumption of fast and processed food, rapid urbanization, and medical technologies, which makes GM more susceptible than in previous years (Sonnenburg and Sonnenburg, 2019).

**Figure 1:**
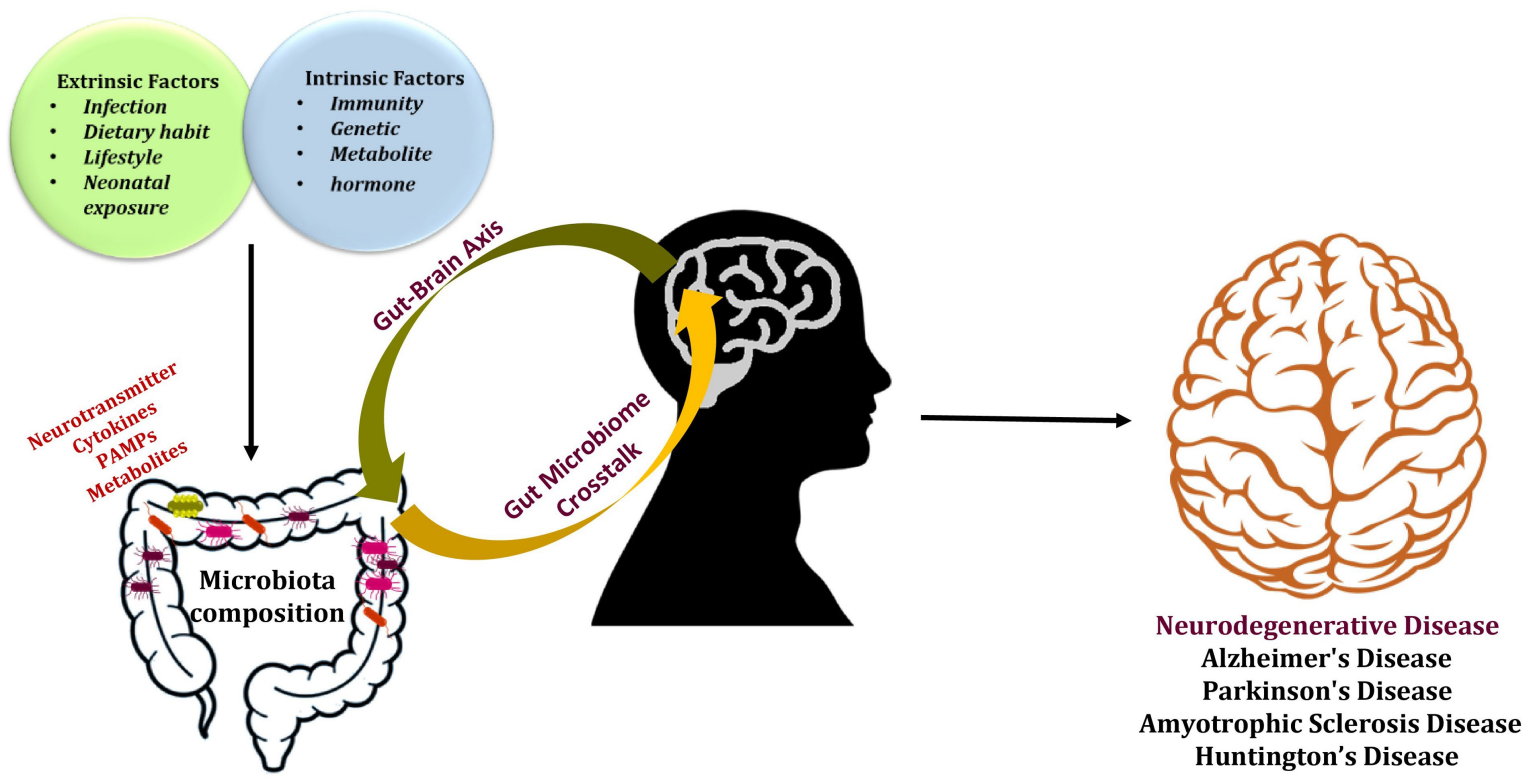
The gut microbiota’s composition is altered by external and internal factors, which also have a role in developing neurodegenerative disorders.

The gut-brain axis (GBA) is a complex communication system that links the gut and the brain. This connection allows bidirectional communication between the gut and brain signals can be sent from the gut to the brain, and vice versa. The GBA plays a significant role in maintaining a human’s general health and wellbeing. It encompasses several pathways, including neural, hormonal, and immune, that work together to regulate various physiology, such as digestion, immune function, and mood regulation (O’Mahony et al., 2015; Dinan and Cryan, 2017). The GM produces a variety of by-products that eventually affect brain’s function through the GBA. These metabolites include short-chain fatty acids (SCFAs), like butyrate, acetate, and propionate. These secreted metabolites exert various functions like modulation of immune function, and influence secretion of neurotransmitters (NTs) like gamma-aminobutyric acid (GABA) and serotonin (5-HT), which participates in regulation of mood, cognition, and anxiety. For example, Kynurenines are synthesized by GM that plays role in modulation of immune response and affecting production of NTs such as glutamate and dopamine. In addition, other NTs like norepinephrine and histamine are also regulated under the influence of GM through the GBA. These metabolites exerts various physiological activities in the brain, including cognitive function, mood, and appetite (Kennedy et al., 2017; Lin and Zhang, 2017; Rowland et al., 2018). A condition known as “dysbiosis,” which is caused by the imbalance in the composition and overall diversity of GM, thus leading to disruption of the normal homeostasis of the gut through the GBA. This causes severe effects on the normal brain activities and affect wellbeing of an individual. The imbalanced state of GM is also responsible for releasing pro-inflammatory cytokines, establishing low-grade inflammatory state in the body, and increasing oxidative stress in the body which leads to cell damage and contributes to aging and disease pathologies. The other detrimental effects caused by the dysbiosis are disruption in the energy metabolism, increased cell death, and modulation of immune response, which contributes to the advancement of various diseases (Noble et al., 2017), adding to the pathogenic mechanisms of several disorders, including neurodegeneration (Mulak and Bonaz, 2015). Dysbiosis has been linked to several other diseases, including: (a) Inflammatory Bowel Disease (IBD): dysbiosis in the GM gut microbiome is associated with IBD, and other gut related disorder like Crohn’s disease and ulcerative colitis (Di Martino et al., 2023); (b) Irritable Bowel Syndrome (IBS): it is associated with dysbiosis, which is characterized by abdominal pain and altered bowel habits (Körner and Lorentz, 2023); (c) Obesity: GM is associated with obesity and has been linked with the dysbiosis due to increased body weight and fat accumulation (Zhang et al., 2023); (d) Type 2 diabetes (T2DM): various studies have demonstrated correlation of T2DM with dysbiosis and stated that may dysbiosis is a contributing factor to T2DM (He et al., 2023); (e) Allergies and Asthma: dysbiosis in the gut and respiratory microbiome has been associated with an increased risk of allergies and asthma (Kleniewska and Pawliczak, 2023); (f) Mental Health disorders: dysbiosis in the gut microbiome has been linked to mental health disorders, including depression, anxiety, and autism (Młynarska et al., 2022); (g) Autoimmune Diseases: dysbiosis in the GM is correlated with autoimmune diseases, including rheumatoid arthritis (RA) and multiple sclerosis (MS; Ordoñez-Rodriguez et al., 2023). In addition, variation in the diversity of GM is associated with aging and aging gut demonstrated a decrease in the microbial diversity (Cheng et al., 2013). Interestingly, elderly individuals with NDs demonstrated a steep change in GM (Claesson et al., 2011). Reportedly, changes in the diversity and composition of GM and dysbiosis is associated with development of brain pathologies like NDs through various mechanisms like promoting inflammation, increased oxidative stress, or disrupted energy metabolism. Few studies suggest that certain gut bacteria can impact proteins, like Aβ, and tau, which are associated with the pathogenesis of NDs. The illustration (Figure 1) represents the dysregulation of GM leading to the development of NDs. Further research is warranted to elucidate the relationship between the GM and NDs. Herein, we have highlighted influence of GM on several NDs and discussed their associated mechanisms, pertinent clinical consequences, and future applications. We have also discussed potential adjuvant therapeutic strategies like probiotics and fecal microbiota transplantation (FMT) for the treatment of NDs.

## 2. Gut microbiota

The gut microorganisms and the host possess a symbiotic relationship, which means they rely on each other for survival. The GM is responsible for various activities like aiding digestion, producing vitamins and other nutrients, and supporting the immune system. In turn, the host offers the GM an appropriate environment and nutrients to survive. This relationship is dynamic and can be influenced by factors such as diet, antibiotics, and stress (Lozupone et al., 2012; Geva-Zatorsky et al., 2017).

Various factors directly influence the diversity and individual variations in the GM. Interestingly, the acidic pH of the gut significantly impacts the different types of microorganisms that survive and thrive in the gut. Moreover, the immune system plays a crucial role in sculpting the curvature of the GM. Factors like genetics and environmental exposure can significantly influence the ability of the immune system to regulate a healthy balance of GM. Interestingly, the digestive enzyme also has a role in shaping the GM. At the strain level, the dynamics of GM is also influenced by other factors like genetics, medical conditions and history, diet and supplements, physical activity, and stress. All these factors contribute to the diversification of the GM and also cause individual variations in the GM (Yatsunenko et al., 2012). Each individual poses a unique microbial community that contributes to developing stable and resilient conditions (Lozupone et al., 2012). *Firmicutes* and *Bacteroidetes* are predominantly present in the gut mucosa of an adult, whereas *Actinobacteria*, *Verrucomicrobia*, and *Proteobacteria* are less common (Eckburg et al., 2005). By secreting microbial substances, including lipids, vitamins, and vital amino acids, the GM has the potential to impact human health directly (Diaz Heijtz et al., 2011; Ma et al., 2019; Needham et al., 2020). These elements affect the normal physiological processes of an individual, such as the gastrointestinal barrier, nutrition breakdown, immunological reaction, and aging-related neurological progress through neuronal, endocrine, and immune pathways (Gonzalez et al., 2011; Kau et al., 2011; O’Toole and Jeffery, 2015; Zhou et al., 2021).

## 3. Mechanisms underlying the effect of gut microbiota on neurodegeneration

### 3.1. BBB dysfunction

The blood–brain barrier (BBB) is a complex, multi-layered structure that separates the brain and spinal cord from the circulating blood. BBB comprises three cellular elements: endothelial cells, pericytes, and astrocytes (Ballabh et al., 2004; Daneman and Prat, 2015). Many essential elements needed for CNS function must flow through the BBB, and it also secretes elements into the brain and blood that are essential for preserving CNS homeostasis. Additionally, the BBB might restrict the entry of chemicals from the gut to the brain (Banks, 2019). Aging increases the vulnerability of BBB, which can cause CNS disorders and inflammation of the cerebrovascular system (Obermeier et al., 2013; Banks et al., 2021; Finger et al., 2022). Microorganism-associated molecular patterns (MAMPs) are crucial for regulating the structural integrity and vital cellular processes of microorganisms (Sellge and Kufer, 2015). Upregulation and downregulation of MAMPs are related to either acute or chronic inflammation, which is related to the etiology of a wide spectrum of neurological illnesses (Skaper et al., 2018). BBB permeability is linked to various microbial compounds, like trimethylamines (TMAs), SCFAs, lipopolysaccharides (LPS), and vitamins (Braniste et al., 2014; Harrington, 2015; Aho et al., 2021). The endocrine and immune systems stimulate by these molecules to protect against inflammatory reactions or neurodegeneration or might directly influence neurons by targeting BBB. The outer membrane of gram-negative bacteria comprises LPS, one of the most researched elements of bacteria that is required to induce immune system activation. The excessive amount of LPS can induce systemic inflammatory response and sepsis (Miller et al., 2005). It was reported that LPS administration in germ-free mice reduced the permeability of BBB (Braniste et al., 2014). Reportedly, *via* microglia activation, LPS induced neuroinflammation in germ-free mice (Wendeln et al., 2018). Propionate, acetate, and butyrate are examples of SCFAs produced when resistant starch and indigestible food fibers are digested by the GM, along with other metabolites like methane and hydrogen (Bach Knudsen, 2015). By influencing cellular processes such as G-protein coupled receptor activation and histone deacetylase activation, SCFAs influence psychological functioning and reduce inflammation. These processes also impact host epithelium integrity, BBB integrity, and neural function (Chen et al., 2018; Dalile et al., 2020). Trimethylamine-n-oxide (TMAO), a gut-derived TMA, is secreted by microbiomes including *Anaerococcus*, *Desulfovibrio*, *Clostridium*, and *Providencia* (Romano et al., 2015). The ability of TMAO to traverse the BBB was demonstrated by its presence in cerebrospinal fluid (CSF; Del Rio et al., 2017). The clinical investigation has reported an increased level of TMAO in the CSF of cognitively impaired AD patients. This clinical investigation might help researchers to develop a therapy for conditions like AD, which is characterized by protein misfolding (Vogt et al., 2018). This can be attributed to neurodegeneration linked to complex mechanisms, including aberrant signal transduction, excitation/inhibition imbalance, synaptic dysfunction, oxidative stress, and protein misfolding. These processes are allied with dramatic alterations in the structure and activity of the BBB (Salmina et al., 2021).

### 3.2. Metabolites

#### 3.2.1. Tryptophan metabolites

Tryptophan metabolism by gut bacteria plays a crucial role in regulating host immune responses, GM, and brain functions. Several gut bacteria can produce tryptophan metabolites, including (i) *Lactobacillus*: This is a common genus of lactic acid bacteria in the human gut. *Lactobacillus* can produce several tryptophan metabolites, including indole-3-lactic acid, tryptamine, and 5-HT. (ii) *Bacteroides*: This is a genus of Gram-negative bacteria that are dominant members of the human GM. Bacteroides can produce several tryptophan metabolites, including indole-3-propionic acid (IPA), indole-3-acetic acid (IAA), and indole-3-aldehyde. (iii) *Clostridium*: This is a genus of Gram-positive bacteria, also a member of the human GM. *Clostridium* can produce several tryptophan metabolites, including IPA, IAA, and tryptamine. (iv) *Escherichia coli*: This is a species of Gram-negative bacteria that are commonly found in the human gut. *Escherichia coli* can produce several tryptophan metabolites, including IAA, indole-3-aldehyde, and indole-3-ethanol.

Tryptophan-kynurenine (TRP-KYN) pathway and its by-products were found to be crucial in the development of neuroinflammation (Tanaka et al., 2021a,b). Tryptophan (TRP), a member of the eight amino acids required by humans, is the sole amino acid with an indole structure and is only supplied by protein in the diet. Food consumption and the activation of three tryptophan metabolic processes affect the amount of free TRP in the body. In contrast to the 5-HT and kynurenine channels, which are indirectly controlled by the microbiota of the gut, the indole pathway is controlled directly. Overall, only a small portion of free TRP is used for protein synthesis and synthesizing neurotransmitters like 5-HT and neuromodulators like tryptamine. In contrast, more than 95% of free TRP is the substrate for the TRP-KYN pathway’s degradation (Le Floc’h et al., 2011; Stone et al., 2013), where a variety of bioactive molecules are produced, including immunomodulators, neuroprotective antioxidants, neuroprotectants, and neurotoxins (Tanaka et al., 2021a). Kynurenine uric acid (KA) and Quinolinic acid (QA) are noteworthy examples of the TRP-KYN pathway’s important intrinsic metabolites. Through NMDA-mediated excitotoxicity, QA can cause neurodegeneration (Braidy et al., 2009), while KA, an innate NMDA receptor antagonist, has neuroprotective properties and can control QA’s neurotoxic effects (Vamos et al., 2009). Numerous studies have shown that the TRP-KYN pathway is essential for both: (i) neurodegeneration and (ii) severe brain damage (Schwarcz et al., 2012). Tryptophan 2,3-dioxygenase (TDO) and indoleamine 2,3-dioxygenase (IDO) are the route’s rate-limiting enzymes, which also trigger the TRP-KYN system and produce neuroactive molecules like QA and KA. In addition, the kynurenine per tryptophan quotient (KYN/TRP) ratio is a marker of IDO/TDO activity. Reportedly, the severity of the cognitive function impairment is directly correlated with the rise in the KYN/TRP proportion (Widner et al., 2000). Furthermore, there is proof that the GM can affect how important enzymes in the TRP-KYN pathway function directly. It is important to note that although the activity of IDO is reduced in the intestine of germ-free mice, it can be returned to normal by populating the intestinal system with microorganisms as soon as the animals are weaned (Wikoff et al., 2009; Clarke et al., 2013). A growing body of research has revealed that in AD animal models, the serum levels of KYN and 3-hydroxykynurenine (3-HK), intermediates of the TRP-KYN pathway are significantly increased; in contrast, the concentration levels of TRP and KA are reduced, which was directly connected to impairment of cognitive performance (Ting et al., 2007). In PD, 3-HK concentrations dramatically rose, whereas KA concentrations fell in the substantia nigra putamen and frontal cortex (Ogawa et al., 1992). However, the pathological progression of HD was also thought to be connected to the TRP-KYN pathway metabolites. Furthermore, compared with the control group, HD patients and transgenic mice had higher amounts of 3-HK and QA in their cortex and neostriatum (Guidetti et al., 2004). However, it is interesting that probiotic therapy modifies KYN levels (Desbonnet et al., 2008). Furthermore, several investigations have demonstrated that 5-HT, an important neurotransmitter, can control cognitive performance and lessen Aβ plaque formation (Cirrito et al., 2011). Noteworthy, the digestive system’s chromaffin cells are responsible for secreting 90% of 5-HT. In addition, the common intestinal bacteria *Enterococcus* and *Escherichia coli* can secrete 5-HT (Cryan and Dinan, 2012). Therefore, by regulating 5-HT synthesis, the GM may have an impact on how the CNS regulates.

#### 3.2.2. Short chain fatty acids

A critical metabolic by-product of the GM is SCFAs. Some of the bacterial genera commonly associated with SCFA production in the gut include *Bacteroides*, *Prevotella*, *Clostridium*, *Ruminococcus*, and *Fecalibacterium*. Propionate, butyrate, and acetate, formed from incompletely digested dietary proteins and carbohydrates, comprises most SCFAs (Hamer et al., 2008). SCFAs formed by the GM mainly depend on the relative abundance of the microbiota subgroups or microbiota makeup. For instance, *Bifidobacteria* species mainly synthesize lactate and acetate while *Firmicutes* microbes primarily create butyrate (Tagliabue and Elli, 2013). Concerning the roles of SCFAs, these tiny molecules take part in the physiological signaling of the intestinal epithelium through FFAR2 (G-protein-coupled free fatty acid receptors 2) and FFAR3. However, SCFAs can also reach the systemic circulation either actively or passively, having a wide variety of physiological consequences (den Besten et al., 2013), involving contribution in the breakdown of lipids and glucose (Todesco et al., 1991; Fushimi et al., 2006; Gao et al., 2009). The CNS’s functionality and growth are both impacted by SCFA. For example, it has been demonstrated that SCFA worsens the motor function in sterile PD mouse (Sampson et al., 2016), nevertheless, they enhanced experimental stroke mouse recovery (Sadler et al., 2020). It has been demonstrated that acetate can cross the BBB and inhibit mice’s urge to eat (Wyss et al., 2011; Frost et al., 2014). Butyric acid is a versatile chemical with beneficial neuroprotective properties that enhances brain health. Butyric acid is a crucial energy-producing substrate that can also boost ATP and mitochondrial respiration rates, inhibit histone deacetylase, and influence the activity of various genes and cellular proteins (Bourassa et al., 2016). Additionally, *via* binding to homologous receptors like GPR43, GPR41, peptide YY (PYY), and Glucagon-like peptide 1 (GLP-1), which have a particular function in intestinal endocrine signaling, SCFAs can promote the release of neuropeptides. Once produced, these peptides impact the control of energy balance by stimulating the vagal and intestinal primary afferent pathways (Kuwahara, 2014).

#### 3.2.3. Neurometabolites

Due to the activation of microbiota, a significant number of neurometabolites are directly produced by gut microbes or mucosal epithelial cells. These neurometabolites encompass NTs that directly affect the CNS, signaling cascades, and other signal transduction pathways, all of which have an impact on the CNS’s normal function either directly or indirectly (Forsythe et al., 2012; Lyte, 2014). For instance, when a sufficient substrate is accessible, *Lactobacillus* and *Bifidobacterium* strains can create significant levels of GABA (Barrett et al., 2012). Neurometabolites from the gut interact with the CNS by activating nearby afferent vagal fibers or exerting a distant endocrine effect. Behavioral changes due to differences in NT levels, such as the enhanced activity of spontaneous motor neurons brought on by increased monoamines, 5-HT, and dopamine concentration in the striatum (Barrett et al., 2012). Furthermore, the management of NDs is affected by this phenomenon since the production of NTs is frequently dysregulated and ultimately causes the onset of the disease.

### 3.3. Modification of the nervous system

The bidirectional communication network comprises the CNS, autonomic nervous system (ANS), enteric nervous system (ENS), and the hypothalamic–pituitary–adrenal (HPA) axis. The vagus nerve may be the most direct link between the GM and the brain because it is connected to enteroendocrine cells (Latorre et al., 2016). The solitary tract’s nucleus receives sensory vagal inputs, which are widely distributed to the cerebral cortex and medulla oblongata (Liu and Forsythe, 2021). 20% of vagus fibers transmit signals from the CNS to periphery organs *via* efferent fibers, and 80% of vagus fibers transmit signals to the CNS *via* afferent pathways (Bonaz et al., 2018). The former is associated with anti-inflammatory reflexes, gastric secretions, and gut motility. Vagotomy, a surgical procedure that involves removing a portion of the nerve, is a reliable tool used in the field to examine gut-brain communication (Liu and Forsythe, 2021). Using vagotomy in animal models showed that particular bacterial strains communicate with the brain *via* the vagus nerve, changing behavior performance (Bravo et al., 2011; Buffington et al., 2016). Through vagus nerve pathways, *Lactobacillus rhamnosus* JB1 ameliorates anxiety-related and depressive-like behavior in mice by modulating the GABAergic system in the brain (Bravo et al., 2011). Vagotomised germ-free animals showed no unique neurochemical or behavioral consequences, indicating that the vagal pathway is a crucial channel for gut and brain communication (Forsythe et al., 2014). Direct neurochemical signals from the GM to the brain and vice versa are produced by the ENS in order to communicate with the CNS *via* the vagus nerve (Heiss and Olofsson, 2019). The limbic system’s HPA axis, which controls memory and emotional reactions, comprises the hypothalamus, hippocampus, and amygdala. Stress or inflammatory cytokines like interleukin (IL)-6 raise levels of corticotropin-releasing factor (CRF) released from the hypothalamus and adrenocorticotropic hormone (ACTH) produced from the pituitary gland, which causes the adrenal gland to produce cortisol that is harmful to the brain (Frankiensztajn et al., 2020). Consequently, the CNS can affect the activity and functions of intestinal cells owing to the interaction between neurological and hormonal communications (Sharon et al., 2016; Kaelberer et al., 2018). Additionally, through controlling gut cells and preserving intestinal metabolic and immunological homeostasis, the GM impacts host health (Smith et al., 2013; Sun et al., 2015; Honda and Littman, 2016). For instance, immune-related intestinal and extraintestinal illnesses are thought to include features such as microbial intestinal dysbiosis and increased intestinal permeability linked to *Clostridium* expansion (Zheng et al., 2020). It is interesting to note that the microbiota also affects the production of NTs and hormones that act on the CNS or ENS directly *via* the vagus nerve or indirectly by entering the circulation, such as dopamine, adrenaline, noradrenaline, 5-HT, glucagon-like peptide-1 (GLP-1), GABA, and peptide YY or their precursor chemicals (Strandwitz, 2018; Huang and Wu, 2021).

### 3.4. Modification of the host immune system

Gastrointestinal homeostasis and the development of the host’s immune system depend on GM health (Sommer and Bäckhed, 2013). Immune signaling is induced when the connection between the immune system and the microbiota is dysfunctional, suggesting consequences for NDs (Fung et al., 2017). Numerous immunological signaling pathways, including the inflammasome signaling pathway, type I interferon signaling pathway (IFN-I), and nuclear factor (NF)-κB signaling system, are regulated by the gut bacteria, according to studies (Ma et al., 2019; Oswal et al., 2021). In a study, caspase-1-, the ASC-, and IL-18 deletion mice models displayed altered microbiota compared to the wild-type mouse model, which is characterized by the presence of *Prevotellaceae* species (Gagliani et al., 2014). Additionally, research suggests that major depressive disorder is linked to an active inflammasome and an increase in pro-inflammatory cytokines, such as IL-6, IL-1β, and IL-18 proteins (Young et al., 2014). IFN can prevent inflammasome signaling in multiple sclerosis (Inoue and Shinohara, 2013). IFN-I is linked to the development of dendritic cells (DCs), the expansion of cytotoxic T cells, and the bidirectional communication between the GM and the host (Giles and Stagg, 2017). However, toll-like receptor (TLR) 3-mediated IFN-I production of intestinal DCs can be induced by symbiotic lactic acid bacteria (Kawashima et al., 2013). Additionally, the signs of amnesia and colitis were lessened after the restoration of GM in a colitis model. The crucial transcription factor contributes to immune response; the elevated NF-κB level with the collaborative expression of TNF-α was identified in the intestinal and hippocampal areas that are connected with amnesia (Jang et al., 2018).

## 4. Involvement of gut microbiota in major neurodegenerative diseases

**Figure 2:**
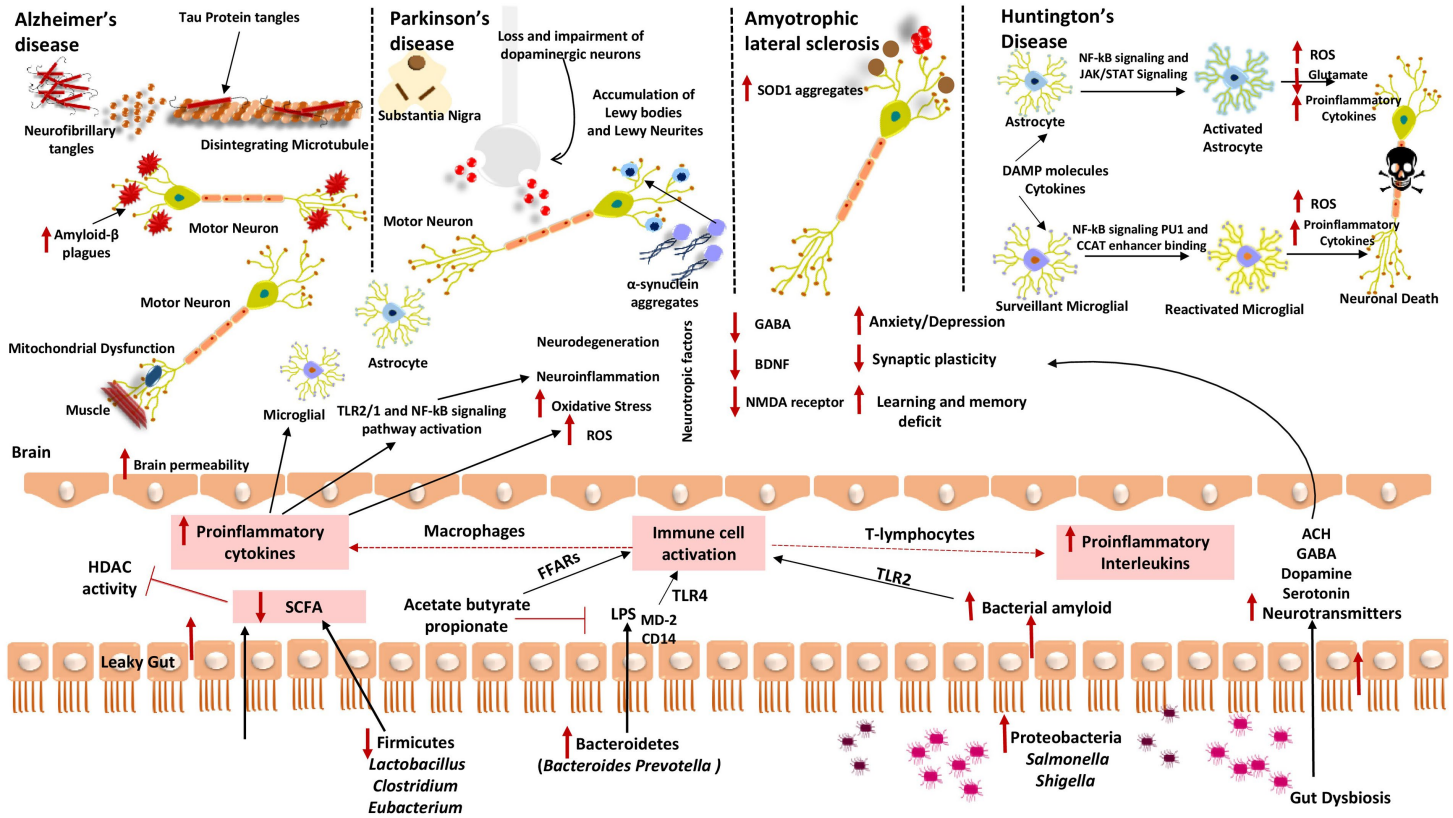
Gut dysbiosis mechanisms underlying several neurodegenerative diseases **(A)** Alzheimer’s disease; **(B)** Parkinson’s disease; **(C)** Amyotrophic lateral sclerosis; and **(D)** Huntington’s disease. When fewer healthy microbes are around to create SCFAs like *Firmicutes* and *Actinobacteria*, HDAC activity and LPS-induced inflammation cannot be inhibited. Contrarily, gut microorganisms like *Bacteroidetes* have a high excretion rate of LPS, which stimulates the TLR4 receptor by interacting with CD14 and MD-2 proteins and results in an inflammatory process. Additionally, proteobacteria produce a lot of bacterial amyloids like curli peptide, which binds to TLR2 to activate macrophages and cause them to release pro-inflammatory cytokines like TNF-α, IL-6, and IL-1β. Additionally, T-lymphocyte activation causes Th17 cells to produce pro-inflammatory interleukins like IL-17A and IL-22. These cytokines can cross the BBB, boost ROS production, and encourage oxidative stress, which results in neuroinflammation and neurodegeneration. These cytokines also stimulate the transcription of pro-inflammatory miRNAs, activate neuroinflammatory mediators, and inhibit microglial cell phagocytosis, which promotes the progression of neurodegenerative diseases. These cytokines also initiate TLR2/1 and NF-kB signal transduction pathways in microglia and astrocytes.

In the coming sections, we explain the mechanisms underpinning how the GM affects the causes of neurodegenerative illnesses (Table 1; Figure 2).

**Table 1:** Involvement of the gut microbiome in neurodegenerative diseases and their GM related plausible mechanisms.

S.no.	Microbiota dysbiosis	Possible mechanism/Pathology	Reference
Alzheimer’s disease (Human studies)			
1.	Increased: *H. pylori*	Patients infected with *H. pylori* with AD showed high cognition impairment. Infection-induced cytokine levels are associated with increased gastric atrophy and linked cognitive impairment, causing tissue damage caused by the increased release of toxins.	Roubaud-Baudron et al. (2012)
2.	Increased: *Escherichia/Shigella* Decreased: *Eubacterium rectale; Bacteroides fragilis*	Increased *Escherichia/Shigella* and decreased anti-inflammatory *Eubacterium rectale* and *Bacteroides fragilis* are associated with increased pro-inflammatory cytokines, which may be correlated with a peripheral inflammatory state in cognitively impaired AD patients. *Bacteroides fragilis* is associated with increased production of lipopolysaccharide.	Cattaneo et al. (2017)
3.	Increased: *K. pneumoniae, B. fragilis, and E. lenta* Decreased: *B. hungatei* and *B. proteoclasticus*	Increased production of pro-inflammatory bacteria and decreased synthesis of butyrate establish potential nexus between AD and intestinal bacteria and modulate intestinal homeostasis with disbalance in pro-inflammatory bacteria and decreased butyrate. Butyrate presents in the colon that maintains the colonic epithelial barrier. In contrast, pathobionts like *K. pneumoniae* could accumulate extracellular amyloids, *B. fragilis* is associated with increased lipopolysaccharide production, and *E. lenta* is associated with severe gastric pathology.	Haran et al. (2019)
4.	Increased: *Oscillospirales* Decreased: *Lactobacillales*	Alteration in the gut microbiota in AD patients is one of the interesting fields. Interaction mechanisms between human microbiota and the brain might reveal interesting findings and uncover novel pathogenesis mechanisms. *Lactobacillales* synthesize antimicrobial peptides known as bacteriocins, which further prevent the growth of pathogens.	Askarova et al. (2021)
Alzheimer’s disease (Animal studies)			
5.	Increased: *Bacteroides fragilis*	*Bacteroides fragilis* is associated with increased production of LPS, which *BF-induced LPS* cross-gut epithelium and enters the bloodstream, inducing a systemic inflammatory reaction and increase of pro-inflammatory cytokines. Increased *Bacteroides fragilis* could be used as a prognostic factor of AD in early life.	Borsom et al. (2023)
6.	Increased: *Proteobacteria Erysipelotrichaceae*	Profile of inflammatory-related bacterial *Proteobacteria* and *Erysipelotrichaceae* might contribute to disease progression.	Bäuerl et al. (2018)
Parkinson’s disease			
7.	Increased: *Verrucomicrobia*, *Mucispirillum, Porphyromonas, Lactobacillus,* and *Parabacteroides* Decreased: *Prevotella*	Increased *Bacteroides* were found in PD patients with non-tremor than with tremor. Hence, *Bacteroides* abundance is associated with disease severity or worsened motor symptoms. Also, increased *Bacteroides* correlated with increased TNF-α and IFN-γ. *Bacteroides* induce inflammation by eliciting pro-inflammatory cytokines and disrupting the gut barrier.	Lin et al. (2019)
8.	Decreased: *Lactococcus spp.*	Decreased population of *Lactococcus spp.* was associated with an increase in lytic phages. A decrease in lactic acid bacteria may disrupt the intestinal barrier.	Tetz et al. (2018)
9.	Increased: *Ralstonia* Decreased: *Blautia, Roseburia,* and *Fecalibacterium*	increased in *Ralstonia* associated with pro-inflammatory bacteria. A decrease in the production of butyrate (SCFA) [*Blautia, Roseburia, Fecalibacterium*] is contributed to gut hyperpermeability.	Keshavarzian et al. (2015)
10.	Increased: *Enterobacteriaceae* Decreased: *Prevotellaceae*	Increased *Enterobacteriaceae* is associated with postural instability and gait difficulties. *Prevotella* synthesis of thiamine and folate. Decreased Prevotella might cause prevention in the synthesis of thiamine and folate.	Scheperjans et al. (2015)
Parkinson’s disease (Animal studies)			
11.	Increased: Proteobacteria Decreased: *Lachnospiraceae and Ruminococceae*	Distribution in the gut microbiota is a hallmark of the motor and GI dysfunction in the mouse model of PD. Disbalance in microbiota causes microbial molecules to instigate gut-brain signaling that may impact neuroinflammation and causes PD pathologies like α-Syn aggregation.	Sampson et al. (2016)
Amyotrophic lateral sclerosis (Human studies)			
12.	Increased: *Bacteroidetes* (Phylum) Decreased: *Firmicutes*	Increased *Bacteroidetes* and decreased *Firmicutes* reflected undermined condition in ALS condition. *Bacteroides* induce inflammation by eliciting pro-inflammatory cytokines and disrupting the gut barrier.	Zeng et al. (2020)
13.	Decreased: *Prevotella spp.*	*Prevotella spp.* Deficiency and modification in butyrate metabolism may have translational value for ALS treatment*—prevotella* synthesis of thiamine and folate. Decreased Prevotella might cause prevention in the synthesis of thiamine and folate.	Hertzberg et al. (2022)
14.	Decreased: *Eubacterium rectale* and *Roseburia intestinalis*	Several butyrate-synthesizing bacteria, which are crucial for gut integrity and inflammation regulation, were reduced in ALS patients compared to healthy controls.	Nicholson et al. (2021)
15.	Decreased: *Firmicutes/Bacteroidetes ratio*	Increased production of pathobionts and reduction in the synthesis of probiotic organisms in the intestine of ALS patients might down-regulated or upregulated production of NO, GABA, LPS, and SCFAs production, which might lead to increased pathogenesis of ALS.	Fang et al. (2016)
Amyotrophic lateral sclerosis (Animal studies)			
16.	Decreased: *Butyrivibrio Fibrisolvens, Escherichia coli,* and *Fermicus*	A decrease in the level of antimicrobial peptides defensin 5-α was found in the ALS intestine, which was correlated with a decrease in the abundance *of Butyrivibrio Fibrisolvens;* this bacterium has a role in the regulation of proteins that regulate the intestinal epithelium permeability.	Wu et al. (2015)
Huntington’s disease (Human studies)			
17.	Increased: *Intestinimonas (genus)* Decreased: *Bilophila*	In systemic inflammatory reactions in HD patients, *Bilophila* may have an anti-inflammatory effect. Pro-inflammatory IL-6 levels had a negative correlation with *Bilophila*. *Intestinimonas* and plasma levels of IL-4, an anti-inflammatory cytokine produced mainly by T-helper-2 cells, are positively correlated.	Du et al. (2020)
18.	Increased: *Bacteroidetes* Decreased: *Firmicutes*	Gut dysbiosis was associated with body weight impairment and motor deficit.	Wasser et al. (2020)
Huntington’s disease (Animal studies)			
19.	Increased: *Bacteroidetes* Decreased: *Firmicutes*	Disbalance in the microbes in the gut/and gut microbiome leads to increase intestinal permeability and causes dysbiosis, which may cause HD pathology.	Stan et al. (2020)

### 4.1. The gut microbiota on Aging

Some significant studies on the GM and normal aging preceded interest in the microbiota and NDs linked to aging. Increased intestinal permeability, inflammation, lowered digestion, and altered nutrient absorption are all frequently associated with aging, and each of these conditions interacts in both directions with the gut flora (An et al., 2018). According to most sequencing research, the makeup of the GM changes from adulthood to old age, which is comparable with these linkages (Langille et al., 2014; O’Toole and Jeffery, 2015). However, there is limited agreement over the specific taxa linked to aging discovered in this research. For instance, some research linked a rise in *Bacteroidetes* with aging (Claesson et al., 2011; Odamaki et al., 2016); in contrast, another study observed increases in *Bifidobacterium*, *Akkermansia*, and *Christensenellaceae* and losses in *Lachnospiraceae*, *Bacteroidaceae*, and *Ruminococcaceae* (Biagi et al., 2016). Several confounding factors likely cause the lack of study consensus, in addition to variations in study design and technical methodologies. For instance, medication intake was linked favorably with *Bradyrhizobium*, *Coprobacter*, *Helicobacter*, and *Prevotella* and negatively with *Massilia* and *Lachnospiraceae* in a study of hospitalized elderly patients and healthy controls (Ticinesi et al., 2017). In a 60-year longitudinal research, married people had more microbial diversity and richness than people who lived alone (Dill-McFarland et al., 2019), which may be connected to findings about how social interaction affects the microbiota in the stomach. Animal models have been beneficial for proving proof of concept for microbial effects on aging due to the technological difficulties of doing mechanistic aging-related research in humans. Changing the GM can alter the lifespan in various fish, flies, worms, and rodents (Gordon et al., 1966; Smith et al., 2017; Obata et al., 2018). Age-related changes in the GM of *Drosophila melanogaster* indicated and preceded age-related intestinal barrier failure and subsequent deterioration (Clark et al., 2015). After exposure to oxidants as children, specific *Acetobacter* depletion from the gut increased longevity (Obata et al., 2018). In contrast, excessive *Lactobacillus plantarum* growth increases reactive oxygen species, which in turn shortens lifespan (Iatsenko et al., 2018). Extending the longevity of African turquoise killifish by transplanting their middle-aged recipients’ microbiota from young donors coincided with the transfection of *Exiguobacterium*, *Psychrobacter*, *Planococcus*, and *Propionigenium* (Smith et al., 2017). In line with age-related decreases in *Akkermansia muciniphila* in mice and humans (Fransen et al., 2017; Sovran et al., 2019), *A. muciniphila* treatment considerably increased lifespan in progerian mice (Bárcena et al., 2019). Many studies indicate that microbial modulation of immunological homeostasis may be implicated, even though little is known about the precise cellular and molecular mechanisms underpinning these numerous events. Germ-free mice infected with the GM of conventional mice that were old but not young showed heightened production of cytokines in the serum and increased intestinal permeability, indicating that aging-associated alterations in the GM increase inflammatory processes (Thevaranjan et al., 2017). In line with this, transplanting young mice’s fecal microbiota restored deficient B cell proliferation in the Peyer’s patches of old mice (Stebegg et al., 2019), demonstrating defenses against immunological dysregulation brought on by aging in young mice’s microbiome. This pioneering research on the microbiome’s functions throughout healthy aging reveals changes in the microbiome that affect lifespan and health, perhaps due to immunological and stress responses. These findings further make way for active research in microbial influences on aging-related neurological conditions, such as AD, PD, ALS, and HD given that aging is a significant risk factor for many NDs.

### 4.2. Alzheimer’s disease

Alzheimer’s disease (AD), the most prevalent degenerative illness, which is characterized by a loss in cognitive abilities, including memory, speech, and problem-solving (Alzheimer’s Association, 2016). Neuroinflammation, Aβ plaque build-up, and neurofibrillary tau tangles are pathological features of AD. The amyloid precursor protein (APP) cleaved form is Aβ (Aβ40 or Aβ42). By self-aggregation, the Aβ polymerizes form fibrils in the CNS, where it further causes inflammatory reactions and neurotoxicity. Recent research has shown that microbial dysfunction and infection play a significant role in the etiology of AD, particularly in the stimulation of neuroinflammation and the production of amyloids. Patients with AD have been found to have elevated levels of Tumor necrosis factor-α (TNF-α), IL-1β, and other neuroinflammation-related variables (Bagyinszky et al., 2017; Wang et al., 2018). Gareau et al. (2011) demonstrated that infection under acute stress may worsen memory loss and dysfunction. Inflammation and amyloid fibrillogenesis are sparked by releasing LPS in the brain (Asti and Gioglio, 2014). The microglia can identify and remove amyloid molecules by using TLR Pathway (TLR4 and TLR2). Noteworthy, TNF-α and NF-κB are activated by signaling of myeloid differentiation primary response 88 (MyD88) from microglial TLR2, which likewise causes the synthesis of Aβ by increasing α- and β-secretase, respectively (Allen, 2016). Anything insoluble, prone to aggregation, and rich in lipoprotein deposit that matches carbohydrate starches is referred to be amyloid (Schwartz and Boles, 2013). Recently, it has been demonstrated that bacterial metabolites and by-products aggravate AD. Curli, tau, Aβ, α-Syn, and prion, bacteria-derived amyloids, can catalyze cross-section and assemble host amyloids in AD patients (Zhou et al., 2021). Despite having different sequences, amyloids like CsgA and Aβ42 can cause brain deposition and set off a series of AD-related pathogenic events (Hill and Lukiw, 2015). According to reports, persistent *H. pylori* infection could cause AD patients to generate inflammatory cytokines and amyloids (Roubaud-Baudron et al., 2012; Bu et al., 2015). The cell model has demonstrated that hyperphosphorylation of tau might be induced by *H. pylori* filtrate (Wang X. L. et al., 2015). Curli fibers, a significant CsgA subunit, are extracellular amyloids found to be produced by *E. coli*. Other amyloids generated by microorganisms include phenol-soluble Modulins by *Staphylococcus aureus*, TasA by *Bacillus subtilis*, Chaplins by *Streptomyces coelicolor*, FapC by *Pseudomonas fluorescens*, MccE492 by *Klebsiella pneumonia*, and CsgA by *Salmonella* spp. (Schwartz et al., 2012; Schwartz and Boles, 2013; Friedland and Chapman, 2017). In fecal specimens from an APP-transgenic mouse, Harach and the group have identified a striking change in the GM and concluded that the microbiota contributed to the formation of this degenerative brain disease. Comparing controls and intestinal germ-free APP transgenic mice, it was discovered that the later had less cerebral amyloid pathology (Harach et al., 2017). More recently, it has been shown that *Chlamydia pneumoniae* invasion in astrocytes contributes to the production of Aβ, which encourages AD (Al-Atrache et al., 2019). It was suggested that the use and implementation of probiotic is helpful in AD. In a mouse model where Aβ was administered, Athari Nik Azm et al. (2018) showed that *Lactobacilli* and *Bifidobacteria* were successful in improving learning and memory deficits. Wang and Liang and colleagues have demonstrated the implementation of *Lactobacillus fermentum* NS9 and *Lactobacillus helveticus* NS8 have improved spatial memory under persistent restraint stress and reduced the symptoms of ampicillin-induced impairment (Liang et al., 2015; Wang T. et al., 2015). Akbari et al. (2016) demonstrated that mini-mental state examination (MMSE) scores significantly improved after 12 weeks of supplementation of *Lactobacillus* and *Bifidobacterium* species through fermented milk in a randomized clinical study of probiotic treatment in 60 AD patients. In addition, glucose and lipid metabolism modifications were noted, which were believed to improve the cognitive evaluation score (Akbari et al., 2016). There were improvements in cognition measures seen in AD patients when *H. pylori* is eradicated with triple treatment (Kountouras et al., 2009). The established studies demonstrated that using probiotics and antibiotics could open a new window in treating AD. More recently, it was demonstrated that probiotics supplementation and exercise could improve cognition function, reduce the amount of Aβ plaques in the hippocampal region, and eventually reduce disease progression in the AD mice model (Abraham et al., 2019).

### 4.3. Parkinson’s disease

More than 1% of the adult population > 65 years of age has PD, which is a common neurodegenerative illness (Reeve et al., 2014). It is hypothesized that PD results from the interaction of environmental and genetic factors. Motor impairments and non-motor symptoms (NMS) are the hallmarks of this neuropathology, which ultimately impacts life quality (Felice et al., 2016). The GM attained more interest by the scientific society because it has a potential connection with the development of PD (Felice et al., 2016). PD is accompanied by several enteral dysfunctions, including malnutrition, infection due to *H. pylori* infection, and constipation (Fasano et al., 2015). A greater incidence of α-syn presence is identified in patients compared with controls from various studies regarding the function of GI tract disease in PD (Chiang and Lin, 2019). Animal studies have established that vagus nerve excision can prevent α-syn from traveling from the gut to the CNS (Ulusoy et al., 2017). Additionally, bowel inflammation can lead to neuroinflammation, which encourages dopaminergic neuron death in the substantia nigra region of rodents (Villarán et al., 2010). Forsyth and co-workers discovered that PD progression is impacted by “leaky intestines” and intestinal inflammation (Forsythe et al., 2014). Increasing *E. coli* and α-syn deposition in PD patients’ sigmoid was observed to be linked with increased colonic permeability (Salat-Foix et al., 2012). While the butyrate-producing and anti-inflammatory bacterial genera *Blautia*, *Coprococcus*, and *Roseburia* are markedly deficient in PD patients, the numbers of the LPS-producing genera *Bacteroides* and *Oscillospira* are noticeably higher (Keshavarzian et al., 2015). *Ralstonia*, a pro-inflammatory genus, is much more prevalent in the mucosa of PD patients, suggesting that the inflammatory gut barrier is harmful. Scheperjans et al. (2015) demonstrated that PD could be diagnosed using the changed gut flora and the associated motor phenotype. *Prevotellaceae’s* relative abundance is markedly decreased in PD, which has been proven to be a particularly profound biomarker for PD diagnosis. 90.3% specificity in diagnosing PD was demonstrated by a model based on the presence of constipation and several bacterial families. PD problems like constipation have been shown to be improved by consuming fermented milk for 4 weeks (Barichella et al., 2016). According to two studies, immunosuppressants and anti-TNF medication lower the risk of PD (Camacho-Soto et al., 2018; Peter et al., 2018). However, there is a severe lack of clinical evidence supporting the probiotics use in treating PD, and more research is required.

### 4.4. Amyotrophic lateral sclerosis

Amyotrophic lateral sclerosis is a progressive ND that results in the death of motor neurons, spinal cord cells, and brain cells. Muscle twitching, cramping, stiffness, coordination issues, and muscle weakness are among the symptoms. This eventually makes it harder to breathe, speak, and swallow. ALS exists in two forms: sporadic ALS, which is the most prevalent kind with an unclear etiology, and familial ALS, which is brought on by gene alterations (Toepfer et al., 1997). The G93A SOD1 (superoxide dismutase) transgenic ALS mouse model is utilized to study the function of GM in the illness. In a preclinical model, three stages can be distinguished in the ALS progression: pre-onset syndrome lasts 60–70 days, the onset lasts 90–100 days, and progressive lasts 120–130 days (Zhou et al., 2015). The transgenic mice exhibit higher intestinal permeability and impaired BBB during the pre-onset period. It has been documented that lower levels of E-cadherin and zonula occludens (ZO)-1 cause gut wall damage. Reduced quantities of butyrate-producing bacteria, such as *Peptostreptococcus* and *Butyrivibrio fibrosolvens* were found in the feces of SOD1G93A mice, which led to an upsurge in serum and intestine inflammatory cytokine, IL-17 (Wu et al., 2015; Fang, 2016). Zhang and co-workers demonstrated that 2% supplementation of butyrate in their drinking water to SOD1 transgenic mice prevented the progression of ALS. The prevention in ALS progression is possible because of a significant increase in the abundance of *B. fibrosolvens*, improving the function of the intestinal barrier (Zhang et al., 2017). Interestingly, fecal sample of ALS patients showed less butyrate-producing bacteria such *Anaerostipes*, *Oscillibacter*, *Lachnospira*, and more *Dorea* species. *Dorea* may produce ethanol as a by-product of glucose metabolism, so it is deemed toxic (Fang et al., 2016). It has also been found that ALS patients’ plasma levels of LPS and β-Methylamino-L-alanine differ. Circulation of this chemical in the bloodstream may enter the CNS and contributes to ALS development by causing brain inflammation and disrupting BBB function (Bengmark, 2013; Catanzaro et al., 2015). BBB disruption is widespread in ALS patients, including humans and animals (Garbuzova-Davis and Sanberg, 2014). Probiotics’ therapeutic potential for treating ALS-like diseases is still being investigated.

### 4.5. Huntington’s disease

Huntington’s disease is an autosomal dominant, uncommon ND with an estimated global prevalence of 2.7 cases per 100,000 people with an onset age between 35 and 44 years (Erkkinen et al., 2018; Gatto et al., 2020). Cognitive impairment, mental problems, and motor symptoms are the three classical pathological signatures of HD. Dysphagia develops from motor disruption, causes loss of weight and aspiration issues, and is lethal (Rosas et al., 2008; Erkkinen et al., 2018).

The prospect of incorporating GM into HD diagnosis and treatment arises from recent research that links GM with neurological health (Wasser et al., 2020). Alterations in gut microbiome diversity or abundance may indicate HD, and these changes may involve variances in biological sex (Du et al., 2020). A study has reported that the microbiota in the gut of HD mouse models compared to wild-type mice found that male HD mouse models had higher abundances of *Bacteroidales* and *Lactobacillales* and lower levels of *Clostridiales*. Female HD mice, on the other hand, had higher levels of *Coriobacteriales*, *Erysipelotrichales*, *Bacteroidales*, and *Burkholderiale* and a lower abundance of *Clostridiales*. In addition, compared to the female and wild-type mice, male HD mice displayed greater microbial diversity (Kong et al., 2020). Subsequently, a study found that microbiota-deficient animals had lower numbers of mature oligodendrocytes and myelin-related proteins in the prefrontal cortex, which inhibited the plasticity of white matter and callosal myelination (Radulescu et al., 2020). This study demonstrated that depletion of the microbiome could exacerbate HD symptoms. In context to the GM association with HD, some SCFAs and bioactive elements secreted by the GM are observed in HD development and progression. These substances primarily affect the GBA’s biological functions (Konjevod et al., 2021). Tyrosine, IPA, 2-hydroxyphenylacetic acid, 3-hydroxyphenylacetic acid, and 4-hydroxyphenylacetic acid can all result in dietary and bioactive compounds dysbiosis in GBA, while 5-HT, tyrosine, these acids, and hydroxyphenylacetic acids can all cause intestinal permeability (Rosas et al., 2015). The intricate communication between the GM and HD will be better understood with more research on these compounds from the GM. This research will also aid with early detection and HD therapeutics.

## 5. Microbiota-based treatments

Probiotics therapy and fecal microbiota transplantation (FMT) are the two main types of microbiota-associated therapy that is being used as adjuvants nowadays to treat neurological illnesses (Abuaish et al., 2021; Table 2).

**Table 2:** Modification of the gut microbiome in preclinical and clinical investigations using various microbiota therapies.

S.no.	Interventions	Models/Subjects	Major findings	Reference
Probiotic administration				
Human studies				
1.	DW2009: a mixture of fermented soybean powder and *L. plantarum* C29 freeze-dried powder	MCI patients	Enhanced cognitive performance, higher blood BDNF levels, and increased Lactobacilli abundance.	Hwang et al. (2019)
2.	*Bifidobacterium bifidum* BGN4 and *Bifidobacterium longum* BORI	Randomized double-blind, multicenter clinical trial. >65 years old (63 healthy elders)	Increased levels of serum BDNF level, mental flexibility, and alleviation of stress.	Kim et al. (2021)
3.	Multispecies probiotics (*Lactobacillus, Lactococcus,* and *Bifidobacterium*)	60–93 years old (20 AD patients)	Increased levels of serum kynurenine concentrations, kynurenine to tryptophan ratios indicates activation of dendritic cells and macrophages.	Leblhuber et al. (2018)
Animal studies				
4.	*Lactobacillus helveticus* R0052 and *Bifidobacterium longum* R0175 *Lactobacillus rhamnosus*	Lipopolysaccharide-induced rats	Reduction of pro-inflammatory cytokines, reduction of apoptosis in the hippocampus, and enhanced memory (increased expression of BDNF).	Mohammadi et al. (2019a,b)
5.	*Lactobacillus rhamnosus* (GG), *Bifidobacterium animalis lactis* (BB-12), and *Lactobacillus acidophilus* (LA-5)	MPTP-induced mice	Butyrate, through upregulating neurotrophic factors and suppressing the level of Mao B, inhibited the death of dopaminergic neurons.	Srivastav et al. (2019)
6.	*Lactobacillus plantarum* C29-fermented defatted soybean (FDS, DW2009)	5XFAD transgenic mice	Suppression of Aβ, β/γ-secretases, caspase-3 expression, and NF-κB activation increased BDNF expression in the brain.	Lee et al. (2018)
7.	*Lactobacillus fermentum* NS9	Ampicillin-induced SD rats	GM proportion returned to normal, and deficits in spatial memory and anxious behavior caused by ampicillin were reversed.	Wang X. L. et al. (2015)
8.	*Bifidobacterium breve* strain A1	Aβ-injected male ddY mice	Increased cognitive function, controlled immunological responses, and decreased brain inflammation.	Kobayashi et al. (2017)
9.	*Lactobacillus plantarum* MTCC1325	D-galactose-induced AD albino rats	Enhanced histopathological in brain regions, acetylcholine levels decreased Aβ plaque formation, and increased cognitive function.	Nimgampalle and Kuna (2017)
10.	*Bifidobacterium breve* CCFM1025	AD mouse model	Enhanced synaptic plasticity, elevated levels of BDNF, fibronectin type III domain-containing protein 5 (FNDC5), and postsynaptic density protein 95 (PSD-95).	Zhu et al. (2021)
11.	ProBiotic-4 (*Bifidobacterium* and *Lactobacillus*)	Aged Senescence-accelerated mouse prone 8 (SAMP8) mice	Decreased levels of IL-6 and TNF-α, Decreased LPS, TLR-4, and NF-κB, abrogation of retinoic-acid-inducible gene-I multimerization.	Yang et al. (2020)
12	Human-origin probiotic cocktail (five *Lactobacillus* and five *Enterococcus*)	C57BL/6 J male mice	Decreased inflammation, leaky gut, gut dysbiosis, and metabolic disorders.	Ahmadi et al. (2020)
Fecal microbiota transplantation (FMT)				
Animal studies				
13.	ADLP^APT^ mice were administrated fresh feces of wild-type mice	(ADLP^APT^) transgenic mouse model of AD	Amelioration of formation of Aβ plaques and neurofibrillary tangles, glial activation and cognitive dysfunction, decreased expression of genes associated with intestinal macrophage activity and inflammatory blood monocytes.	Kim et al. (2020)
14.	Tg + FMT administrated were administered feces from WT mouse pellets.	APPswe/PS1dE9 transgenic (Tg) mice and wild-type mice	Decreased levels of Aβ40 and Aβ42, tau protein phosphorylation, decreased levels of COX-2 and CD11b, Increased synaptic plasticity (evident from synapsin I postsynaptic density protein 95 (PSD-95) and expression).	Sun et al. (2019)

### 5.1. Probiotics

Numerous studies have demonstrated that implementing beneficial bacteria and probiotics effectively prevents and treats NDs. Oral delivery of probiotics as a cocktail of strains or with a single strain has demonstrated promising results as effective adjuvant therapy. In Probiotic-4, for instance, *Lactobacillus casei*, *Bifidobacterium lactis*, *Lactobacillus acidophilus*, and *Bifidobacterium bifidum* are all present. By suppressing the NF-κB signaling cascade and the inflammation, which RIG-I and TLR4 regulate, it acts to considerably inhibit *Pseudomonas*, *Proteobacteria*, and the *Lachnospiraceae* NK4A136 group; also, it decreases the proportion of *Firmicutes* to *Bacteroidetes* and enhances cognitive performance in a significant manner in SAMP8 mice (aged Yang et al., 2020). In another study, rats were given (receiving Aβ injections for 8 weeks), a probiotic mixture of *Lactobacillus fermentum*, *Lactobacillus acidophilus*, *Bifidobacterium longum*, and *Bifidobacterium lactis*. The altered elements of the GIT microbiota were found to be accompanied by enhanced spatial memory, learning disabilities, and a decrease in reactive oxygen species, according to the research (Athari Nik Azm et al., 2018). The microbiome of 3xTG AD mice was recently shown to be modulated by SLAB51, a preparation of *Bifidobacterium* and *Lactobacillus*, boosting the number of *Bifidobacterium* spp. while decreasing the *Campylobacter* population. The investigational study concluded that these changes in the composition of the microbiota, along with the large quantities of SCFAs in the intestine and the raised amount of the intestinal neuroprotective peptide hormone, may lessen cognitive dysfunction by reduction of Aβ aggregates and, consequently, neuron injury (Bonfili et al., 2017). By expressing BDNF proteins, *Bifidobacterium longum* R0175 and *Lactobacillus helveticus* R0052 were able to reduce the inflammatory mediators levels in a significant manner that were brought on by LPS in the serum and hippocampus, lowering the apoptotic cell death of hippocampus, and lessening the detrimental effects of LPS on memory (Mohammadi et al., 2019a,b). In a different study, *Bifidobacterium breve* A1 was administered to mice who had received an injection of Aβ; this treatment enhanced the mice’s behavior and cognition while suppressing the immune reaction and expression of genes responsible for inflammation in the hippocampal region (Kobayashi et al., 2017). According to a different study, 60 days of therapy with *Lactobacillus plantarum* MTCC1325 in a rat model of D-galactose consumption reduced cognitive impairment and returned acetylcholine levels and histopathological characteristics to normal levels (Nimgampalle and Kuna, 2017). According to studies, *Lactobacillus helveticus* NS8 and *Lactobacillus fermentum* NS9 improved spatial memory under persistent restraint stress and reduced the symptoms of ampicillin-induced impairment (Liang et al., 2015; Wang T. et al., 2015). Recently, a study also demonstrated that a probiotic combination and physical activity could lessen Aβ plaque deposition in the hippocampal region, enhance memory function, and eventually delay AD onset in an APP/PS1 model of mice (Abraham et al., 2019). According to another study, G93A mice’s intestinal epithelial barrier integrity and GM balance were restored following a 2% butyrate treatment, a naturally occurring product from bacteria that aids in restoring the homeostasis of the gut microbiome. In addition, G93A mice showed extended survival and decreased body weight in addition to alleviating the disease’s peripheral and central symptoms (Zhang et al., 2017). *Bifidobacterium* and *Lactobacillus* are frequently employed in probiotic formulations as these bacteria are frequently used to boost people’s condition and are classified as GRAS (generally regulated as safe) for the consumption of humans (Fijan, 2014). In another study, AD patients received 200 mL/D of *Lactobacillus acidophilus*, *Lactobacillus fermentum*, *Bifidobacterium bifidum*, and *Lactobacillus casei* for 12 weeks. Probiotic implementation resulted in positive impact on cognitive performance of AD patient and insulin metabolism but no discernible impact on inflammation, oxidative damage, fasting blood glucose, or lipid distribution compared to the control group (Akbari et al., 2016). Nevertheless, according to Agahi et al. (2018), the serum inflammatory molecules: IL-6, TNF-α, and IL-10, oxidative stress markers: glutathione (GSH), nitric oxide (NO), and malondialdehyde (MDA), did not significantly change after patients with AD received *Lactobacillus plantarum*, *Lactobacillus fermentum*, *Bifidobacterium lactis*, or *Lactobacillus acidophilus*, *Bifidum*, and *Longum* for 12 weeks. As a result, the dosage and formulation of the administered probiotics are only one factor in determining how effective probiotic intervention will be for AD patients. The severity of the disease itself has a significant impact. The results of a Repeatable Battery for the Assessment of Neuropsychological Status (RBANS) and Mini Mental State Examination (MMSE) showed significant changes after 12 weeks of administration of *Bifidobacterium breve* A1 capsule, while shifts in the serum lipid levels, inflammatory mediators, and oxidative stress were insignificant. This suggests that *Bifidobacterium breve* A1 is secure and can enhance reduced cognitive abilities (Kobayashi et al., 2019). Additionally, it has been demonstrated that consuming fermented milk for 4 weeks can reduce the severity of PD problems, especially the symptoms of constipation (Barichella et al., 2016). Numerous research showing the crucial functions endogenous bacteria play in our health, particularly concerning neurodegeneration, provided compelling evidence for the safe use of probiotics (Table 2). These investigations have demonstrated that using particular microbial strains as probiotics could provide favorable outcomes.

### 5.2. Fecal microbiota transplantation

Fecal microbiota transplantation (FMT) is the method of transferring GM from the healthy donors’ from their feces into dysbacteriosis patients’ intestines through enema, nasogastric tube, nasointestinal tube, or endoscopic methods to return the gut microbiome to its healthy biodiversity and functioning (Staley et al., 2017; Parker et al., 2020). This approach is thought to be a successful remedy for *Clostridium difficile* infections that come back several times (Gulati et al., 2020). FMT is regarded as a viable adjuvant therapeutic for some extraintestinal disorders, particularly NDs, due to the GBA’ bidirectional signal connection. For instance, fecal microbiome from APP/PS1-21 male mice was transplanted into age-matched, antibiotic-treated APP/PS1-21 mice, and Dodiya et al. (2019) discovered that this restored the normal GM and partly reversed Aβ disease and microglia architecture. Another investigation discovered that mice transplanted with feces from AD patients had worsened cognitive abilities and less fecal metabolites linked to the neurological system than healthy control mice (Fujii et al., 2019). Clearly, there are not many studies on how FMT affects neurological disorders in people right now, and safety is still a big problem for applying FMT research to human trials. Fecal samples and donors from FMT are tested for potential pathogenic viruses, bacteria, and various other microorganism just like in past or present human investigations (Woodworth et al., 2017). However, the precise and ideal microbial makeup of the transplantable samples is still being studied, which, in terms of human trials, creates possible safety questions and makes it difficult to evaluate results.

## 6. Conclusion

The GM has role in sustaining brain physiology and intestinal flora and particular field received attention by medical fraternity lately. However, there are various challenges that are associated with the current research on GM. The GM controls neurotransmission and vascular barriers *via* the immune function and microbial metabolites, influencing host cognitive function and cerebral vascular physiology. The plausible identified treatment is currently under investigational and developmental phase. The experimental design, individuals, models, analytical technique, pipelines, and quality assurance protocols employed in metabolomics investigations continue to present difficulties. Evidence has shown correlations, not causative links, between the host’s physiology and the microbiota. More sample-size research is required for metagenomics biomarker screening because human feces experiments have many confounding factors. When exposure, demography, food, and socioeconomic conditions are considered, such research are more trustworthy. Although the outcome variables in population-based metagenomics investigations are substantially affected by confounding and irreducible variables, the microbiota composition may be described by a relatively limited number of variables (Falony et al., 2016; Rothschild et al., 2018). Another challenge is the moderate and long-term impacts on cognitive or psychological function identified in contemporary microflora translational medicine. Microbiota-based treatments may take months or years to impact neuropsychiatric illnesses, but their impact on host coagulation can be seen much more quickly. Animal models, alongside human cohort and cross-sectional research, offer crucial data that help to determine how particular microorganism impact the host. In order to verify the roles of specific microbial species, preclinical experiments frequently use animal models, particularly rodents. The initial animal studies aimed to identify relationships between host physiology and the impact of the microbiome in germ-free species (Jonsson and Bäckhed, 2017). The specific characteristics and actions of the microbiota are mostly understood through probiotics, specialized bacteria that colonize germ-free animals, and FMT techniques. Antibiotic-treated animal models are alternatives to evaluating the microbial depletion effects on mature immune wild-type animals (Knight et al., 2018). Mice have a significantly small intestine and larger cecum than humans have in terms of intestinal structure. The surface of mouse mucosa is smoother, in contrast to the human small intestine, which has more circular folds that increase its surface area (Scholtens et al., 2012; Hugenholtz and de Vos, 2018). As animals and humans have dissimilar life histories, the routine of physical activity, circadian rhythm, social constraints, and food composition, life experiences come up during data processing. However, in human research, quantitative cohort research has provided approval than retrospective questionnaires, and sometimes, lower sample numbers are sufficient to test an idea (Zhu et al., 2017). Additional significant problems are the choice of analysis of microbiome methodologies and the associated technical stability (Costea et al., 2017). The microbiota-based therapeutics is a probable strategy to be applied in treatments for brain related diseases in the near future, although being far from perfection.

## Author contributions

SK, NK, SR, and GB carried out the literature review, and drafted the manuscript. SK and NK conceptualized, edited, and provided necessary suggestions, provided critical revisions, contributed to the final manuscript, and also designed the figures. All authors contributed to the article and approved the submitted version.

## Conflict of interest

The authors declare that the research was conducted in the absence of any commercial or financial relationships that could be construed as a potential conflict of interest.

## Publisher’s note

All claims expressed in this article are solely those of the authors and do not necessarily represent those of their affiliated organizations, or those of the publisher, the editors and the reviewers. Any product that may be evaluated in this article, or claim that may be made by its manufacturer, is not guaranteed or endorsed by the publisher.

## Glossary

3-HK: 3-Hydroxykynurenine
5-HT: Serotonin
ACTH: Adrenocorticotropic hormone
AD: Alzheimer’s disease
ALS: Amyotrophic lateral sclerosis
ANS: Autonomic nervous system
APP: Amyloid precursor protein
Aβ: Amyloid beta
BBB: Blood brain barrier
BDNF: Brain derived neurotrophic factor
CNS: Central nervous system
CRF: Corticotropin-releasing factor
CSF: cerebrospinal fluid
DCs: Dendritic cells
ENS: Enteric nervous system
FFAR2: G-protein-coupled free fatty acid receptors 2
FMT: Fecal microbiota transplantation
GABA: Gamma-aminobutyric acid
GBA: Gut-brain axis
GIT: Gastrointestinal tract
GLP-1: Glucagon-like peptide
GM: Gut microbiota
GRAS: Generally regarded as safe
GSH: Glutathione
HD: Huntington’s disease
HPA: Hypothalamic–pituitary–adrenal axis
IAA: Indole-3-acetic acid
IBD: Inflammatory bowel disease
IBS: Irritable bowel syndrome
IDO: Indoleamine 2,3-dioxygenase
IFN-1: Type I interferon
IL: Interleukin
IPA: Indole-3-propionic acid
KA: Kynurenine uric acid
LPS: Lipopolysaccharides
MAMPs: Microorganism-associated molecular patterns
MDA: Malondialdehyde
MMSE: Mini-mental state examination
MyD88: Myeloid differentiation primary response 88
NDs: Neurodegenerative diseases
NF-κB: Nuclear factor-κB
NMS: Non-motor symptoms
NO: Nitric oxide
NTs: Neurotransmitters
PD: Parkinson’s disease
PYY: peptide YY
QA: Quinolinic acid
RBANS: Repeatable battery for the assessment of neuropsychological status
SCFAs: Short-chain fatty acids
SOD: Superoxide dismutase
T2DM: Type 2 diabetes mellitus
TDO: Tryptophan 2,3-dioxygenase
TLR: Toll-like receptor
TMAO: Trimethylamine-n-oxide
TMAs: Trimethylamines
TNF-α: Tumor necrosis factor-α
TRP: Tryptophan
TRP-KYN: Tryptophan-kynurenine
α-syn: α-Synuclein
